# Retraction Note to: Antimicrobial misuse in pediatric urinary tract infections: recurrences and renal scarring

**DOI:** 10.1186/s12941-021-00425-y

**Published:** 2021-03-25

**Authors:** Jayaweera Arachchige Asela Sampath Jayaweera, Mohommed Reyes

**Affiliations:** 1 Department of Microbiology, Faculty of Medicine and Allied Sciences, Rajarata University of Sri Lanka, Saliyapura, Sri Lanka; 2 Department of Pediactrics, Faculty of Medicine and Allied Sciences, Rajarata University of Sri Lanka, Saliyapura, Sri Lanka

## Retraction Note to: Ann Clin Microbiol Antimicrob (2018) 17:27 10.1186/s12941-018-0279-4

The Editors-in-Chief have retracted this article. Contrary to the statement in the article, the authors subsequently stated that they did not obtain ethics approval for this study. Both authors agree with this retraction.

